# OLDER AGE PREDICTS POSITIVITY IN HOSPICE FAMILY CAREGIVER AUDIO DIARIES: IMPLICATIONS FOR ASSESSMENT INNOVATION

**DOI:** 10.1093/geroni/igae098.2030

**Published:** 2024-12-31

**Authors:** Kristin Cloyes, Megan Thomas Hebdon, Lee Ellington

**Affiliations:** Oregon Health & Science University, Portland, Oregon, United States; The University of Texas Austin, Austin, Texas, United States; University of Utah, Salt Lake City, Utah, United States

## Abstract

The likelihood of becoming a caregiver of someone with a serious illness increases with age, and those 55 years of age and older are more likely to be end-of-life caregivers of family and friends. Older adults may also use more emotional coping skills, which can have a protective effect depending on perceived stress and gender. We asked in-home hospice family caregivers of cancer patients (n=58) to record daily, open-ended audio diaries about their experiences. We examined whether age and gender predicted the amount of affect-related content and overall emotional tone (positivity/negativity). Caregivers recorded audio diaries up to 5 minutes via an IVR system (n=984 entries). We transcribed diaries and used LIWC software to quantify the proportion of affect-related content and positive/negative tone, and mixed-effects regression modeling to analyze the effects of age and gender on these indicators. Our sample (M age=60.72, SD=13.12, range=30-86) was mostly white (84.5%) non-Hispanic (86.2%) women (74.1%) caring for their spouse (44.8%). Age predicted positivity, with each additional year of age predicting a slight increase (β=0.426, p=0.028). Age did not predict affect (β=0.032, p=0.251). Gender did not predict tone (β=2.622, p=0.619), but trended towards predicting less affect expression in men (β= -1.482, p=0.062). Hospice family caregiving is challenging and often isolating, affording few opportunities for processing experiences. Our findings suggest an interplay of age and gender in caregiver self-expression. Understanding how caregivers’ self-expression aligns with underlying coping and unmet needs is important for developing audio diaries as a low-cost assessment of coping and well-being.

